# Metallic nano-particles for trapping light

**DOI:** 10.1186/1556-276X-8-65

**Published:** 2013-02-07

**Authors:** Yongan Tang, Branislav Vlahovic

**Affiliations:** 1 Department of Physics, North Carolina Central University, Durham, NC, 27707, USA

**Keywords:** Nano-particles, Surface plasmons, Optical absorption enhancement

## Abstract

We study metallic nano-particles for light trapping by investigating the optical absorption efficiency of the hydrogenated amorphous silicon thin film with and without metallic nano-particles on its top. The size and shape of these nano-particles are investigated as to their roles of light trapping: scattering light to the absorption medium and converting light to surface plasmons. The optical absorption enhancement in the red light region (e.g., 650nm) due to the light trapping of the metallic nano-particles is observed when a layer of metallic nano-particle array has certain structures. The investigation of the light with incident angles shows the importance of the coupling efficiency of light to surface plasmons in the metallic nano-particle light trapping.

**PACS:**

73.20.Mf, 42.25.s, 88.40.hj

## Background

There has been a growing interest in developing thin film silicon solar cells that minimize material costs and maintains high efficiency. It is because that silicon is an abundant element with almost optimal band gap and excellent junction formation characteristics, and the availability of nano-technologies makes it possible to fabricate high quality desired nano-structures. Currently, most of the solar cells are based on the crystalline silicon wafer with the thickness between 200 and 300 μm, and therefore, around 40% of the cost is the silicon wafer. Scientists proposed to develop the thin film solar cells to save the cost by decreasing the thickness of the silicon. Moreover, there is another reason to develop thin film solar cells beyond the cost, it is the absorption efficiency. Since the minority carrier diffusion length is less than or around 300 nm for amorphous silicon [1-3], it is possible to improve the absorption efficiency for a thin film solar cell. Hydrogenated alloy of amorphous silicon (a-Si:H) has higher absorption coefficient than that of the crystalline silicon. Due to this fact, in the visible part of the solar spectrum, a-Si:H absorbs almost 100 times more than crystalline silicon. In practice, the thickness of a-Si:H solar cells can be around 0.3 μm only [4]. However, a limitation in all thin film solar cell technologies is that absorbance of red spectrum is too small, because of the indirect band gap of silicon. Therefore, one of the major driving forces in the thin film solar cell field is to structure the light-trapping (LT) schemes in order to increase absorption in the red spectrum. One traditional method is to create surface structure on top of the solar cells. However, those surface structures that were used for LT in wafer-based cells are not suitable for thin film solar cells. Since those structures were mostly pyramids with a size of 2 to 10 μm etched into the surface, they are too thick and too large for the thin film solar cells, even the wavelength-scale texture on the substrate followed by thin film solar cell on top are not suitable for thin film solar cells either.

In order to overcome these LT problems and to increase light absorption, new method based on excitation of surface plasmon [5] resonance via scattering from noble metal nano-structures was proposed by Catchpole and Polman [6]. The enhancement of optical absorption and photocurrent in a semiconductor (e.g., crystalline Si) via the excitation of surface plasmon resonances in spherical Au nano-particles deposited on the semiconductor surface was reported [7]. These enhancement in absorption within the crystalline Si results in increased photocurrent response in Si *pn* junction diodes over wavelength ranges that correspond closely to the nano-particle plasmon resonance wavelengths. The application of surface plasmon resonance on a-Si:H was reported [8] in 2006, the forward scattering surface plasmon polariton modes in Au nano-particles deposited above the amorphous silicon film improve transmission of electromagnetic radiation, and an enhancement in short-circuit current density and energy conversion efficiency in amorphous silicon p-i-n solar cells is observed. A method of enhancing light trapping by tuning localized surface plasmons through the modification of the local dielectric environment of the particle was reported [9] in 2009. The surface plasmon resonances can be redshifted by up to 200 nm through the modification of the local dielectric environment of the particles; the optical absorption is increased in an underlying Si wafer fivefold at a wavelength of 1,100 nm and enhances the external quantum efficiency of thin Si solar cells by a factor of 2.3 at this wavelength. The resonance frequency of metal nano-particles depends on the size, shape, particle material, and refractive index of the surrounding medium [10]. The resonance frequency gets redshifted when the dielectric functions of the surrounding medium increase. Mokkapati et al. [11] presented their method for optimizing the light-trapping efficiency of periodic grating arrays of metal nano-particles for Si solar cell applications. They suggest that the pitch of the grating should be chosen to allow at least one diffraction mode propagating outside the escape cone in Si for long wavelength light.

**Figure 1:**
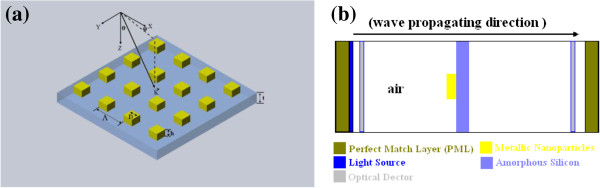
**The schematic diagrams.** (**a**) The metallic nano-blocks on the a-Si:H thin film; (**b**) the cross-section view of the unit cell for simulations.

In this paper, we present the investigation on the LT by the metallic nano-particles as the light scattering elements on a-Si:H thin film (shown in Figure 1). We investigate how the shapes and the sizes of the metallic nano-particles and the periodicities of the unit cells will influence the optical absorption. Our study shows that the optical absorption in the a-Si:H thin film can be significantly enhanced with optimized parameters of nano-particle array.

## Methods

### Surface plasmons and Mie scattering

Surface plasmons are at the interface between a metal and a dielectric material, and have a combined electromagnetic wave and surface charge. They are trapped on the surface, owing to the interaction between the light wave and the free electrons of the metal; the free electrons respond collectively by oscillating in resonance with the light wave. They are transverse magnetic, and the generation of surface charge requires an electric field normal to the surface. This combined character leads to the field component perpendicular to the surface being enhanced near the surface [12]. Metallic nano-particles are strong scatters of light at a wavelength near the plasmon resonance. This is due to collective oscillation of the conducting electron in the metal. As metallic nano-particles are placed on the surface of a thin film (e.g., silicon), these metallic nano-particles will act as light scattering elements; thus, light is trapped in the thin film by scattering from metal nano-particles. Moreover, when these nano-particles are placed close to the interface between two dielectrics, light will be scattered preferentially into the dielectric with larger permittivity. Optical path length in a solar cell is prolonged due to multiple and high angle scattering. The wave vector of the surface plasmon can be described as in the equation below:

(1) $$k_{\text{sp}}=k_{0}\sin\theta +n*\frac{2\pi }{d},$$

where the first term on the left hand side is the part convert from the incident light, *k*_0_ is the wave vector of it, *θ* is the incident angle of the light, the second term on the left hand side is the momentum from the periodic structure, *n* is the refractive index, and *d* is the period of the structure.

Mie scattering theory [13,14] can be applied to find the optimal size of the particles, which will provide larger scattering cross-section than its geometric cross-section and even more importantly larger than its absorption cross-section. The Mie scattering is a scattering of electromagnetic waves by a sphere of radius *a* and permittivity *ε* in homogeneous systems. The scattering and absorption cross-sections are very important because they give the power that is scattered by the particle or absorbed by the particle. The scattering cross-section multiplied by the power density of the incident wave is equivalent to total amount of energy removed from the electromagnetic wave due to scatter in all directions, and a certain amount of energy is absorbed, which results in a heating of the target. The cumulative effective of scattering and absorption is the absorption cross-section.

The scattering efficiency is described as $Q_{s}=\frac{\sigma_{s}}{\sigma_{g}}$, where *σ*_g_ = *πa*^2^ is geometric cross-section and *σ*_s_ is the scattering cross-section; it can be expressed as Equation 2:

(2) $$\sigma_{s}=\frac{2\pi a^{2}}{\alpha^{2}}\sum_{n-1}^{\infty }\;\left(2n+1\right)\left({\left|a_{n}\right|}^{2}+{\left|b_{n}\right|}^{2}\right),$$

where *α* = 2*πa*/*λ*, *λ* is the relative scattering wavelength *λ* = *λ*_0_ / *m*_0_ where *λ*_0_ is the incident wavelength and *m*_0_ is the refractive index of the surrounding medium; *a*_*n*_ and *b*_*n*_ represent the magnetic and electric multipoles of order *n*, respectively. The extinction efficiency is described as $Q_{e}=\frac{\sigma_{e}}{\sigma_{g}}$, where *σ*_e_ is the extinction cross-section; *σ*_e_ = *σ*_a_ + *σ*_s_ is the total cross-section of the particle, and it is described in Equation 3:

(3) σe=2πa2α2∑n=1∞2n+1−Rean+bn.

Therefore, the absorption efficiency is $Q_{a}=\frac{\sigma_{a}}{\sigma_{g}}=Q_{e}-Q_{s}$. We study the size of the particles as a function of the scattering and absorption efficiency using the Mie scattering theory. One important thing to mention is that these higher plasmonic modes are followed by higher absorption which is in accordance with the observations made by [9].

### Metallic nano-particles for LT

We calculated the efficiencies of scattering and absorption of the gold spherical particles in different sizes using the MiePlot (Philip Laven, Geneva, Switzerland) [15]. In this calculation, we choose the sounding medium of air temperature at 25°C and the incident plane wave wavelength from 240 to 840 nm. Our study shows that for a particle with a diameter of 10 nm, which is small when compared with the wavelength, the power scattered by the particle is much less than the product of geometric cross-section and incident Poynting vector. Therefore, the scattering cross-section is much less than geometric cross-section. In other words, the efficiency of absorption is greater than the scattering efficiency of this small particle; thus, for metallic spherical nano-particle, much smaller than an incident wavelength absorption is dominant. Our calculations show that its absorption still prevails over scattering for particles with a diameter of 50 nm, but they are at the same order of magnitude (*Q*_s_ ≈ 6.5 and *Q*_a_ ≈ 7.8) and within a narrow spectrum from 350 to 400 nm. For particles with a diameter of 100 nm, the scattering cross-section is higher (*Q*_s_ ≈ 8 and *Q*_a_ ≈ 2). In this case, the scattering prevails over absorption, and more importantly, the spectrum of the scattering is much broader (300 to 500 nm) than the 50-nm diameter particles; meanwhile, the absorption spectrum is broader. This is because the higher-order plasmon modes are excited. Therefore, the higher plasmonic modes are followed by higher absorption, which is accordance with the observations in [11]. Particles with diameters of 200 and 300 nm are investigated, too. Both particles show similar pattern with broadening the spectrum to the red light wavelength of *Q*_s_. These calculations show that the metallic nano-particle will have a broad spectrum of scattering for particles with a diameter larger than 100 nm; therefore, it is possible to enhance the absorption over a broad spectrum when the solar cell is placed beneath the metallic particles. Moreover, besides the scattering from the metallic nano-particle to the thin film, the surface plasmon of the metallic nano-particles can trap the incident lights to the thin film, too. Thus, the thin film solar cell absorption is enhanced by the metallic nano-particles in two ways: surface plasmons and scattering.

The LT of a thin film of a-Si with metallic nano-particles on its top is investigated. The metallic nano-particles are patterned on the a-Si thin film as shown in Figure 1a, where ***Λ*** is the period of the array; *D* and *h* are the side length and the height of the nano-block, respectively; *t* is the thickness of the a-Si thin film. We choose gold as the metal in this investigation; its optical properties are described by a dispersive complex dielectric function [16], and the optical properties of the a-Si are taken from Sopra N&K Database (Sopra Group, Belfast, Ireland). We applied the finite difference time domain (FDTD) software of MEEP [17] to simulate the metallic nano-particles on a-Si thin film. The sketch of the unit cell for the FDTD is shown in Figure 1b. A plane wave impinges on the metallic nano-particle array with an incident angle of *θ*. The orientation of the incidence plane is located by the azimuthally angle *φ* measured from the *x*-axis. In the simulation, the metallic array is illuminated with the plane wave normal to the metal film (at *θ* = 0 and *φ* = 0).

In these simulations, the a-Si:H thin film is sitting in the middle of a computing unit cell (shown in Figure 1b), the metallic nano-particle is placed on the a-Si thin film, and the boundary conditions of the unit cell are set as periodically (Bloch-periodic in both *x* and *y* directions). Two perfect match layers (PMLs) are put at both ends (*z* direction) in the unit cell. Next to the PML on the right side, a plane wave source is set to illuminate the thin film with metallic nano-particles on it, and two detectors are put into the unit cell to measure the transmission spectra by computing the fluxes of these Fourier-transformed electric fields. It is important to setup proper thickness of the PMLs to reduce numerical reflection. The thicknesses of the PMLs are dependent on the working wavelength.

**Figure 2:**
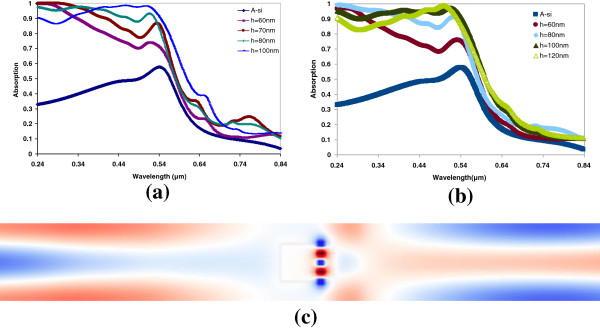
**Absorption enhancement by nano-block and nano-cylinder.** (**a**) Absorption enhancement by nano-blocks as a function of wavelength; (**b**) absorption enhancement by nano-cylinders; (**c**) electric field distribution shows that the metallic nano-cylinder (nano-block has similar effect) particle has a significant effect on trapping light underneath it (incident wavelength at 650 nm).

The absorption spectra of 100-nm a-Si:H thin film without and with metallic nano-particles on its top are shown in Figure 2. The shapes of the nano-particles are very important in the absorption enhancement. Nano-block and nano-cylinders are good for scattering and surface plasmon inducing, but other shapes such as pyramids, cones, hemispheres, and spheres are not as good from the theoretical prediction, some have less surface plasmon-inducing ability and some do not have good scattering effect. The optical absorption of the a-Si:H thin film with particles of nano-blocks and nano-cylinders are shown for Figure 2a,b. The nano-blocks are 100 × 100 nm × *h*, and the nano-cylinders' radii are 50 nm. The reason to choose a square (or circle) base is that the sides of the square have equal ability to induce surface plasmons from all polarizations of the incident sunlight. The periodicity is set as 200 nm, in other words, that 25% of the thin film is covered by the particles in the nano-block configuration, and about 19.6% of thin film is covered by particles in the nano-cylinder configuration. It shows that the LT is hard to observe in the red light region for *h <* 50 nm, and the optical absorption efficiency is improved drastically for the short wavelength light. However, our focus is on the improvement in the red light region. Both nano-block and nano-cylinder show significant increase of absorption efficiency for 100-nm high particles. The electric field distribution of the metallic nano-cylinder on a-Si:H thin film is shown in Figure 2c. It shows that there is incident light trapped under the particles, and the light loss due to ohmic loss in the metal is very limited compared to the enhancement of the absorption in the thin film.

**Figure 3:**
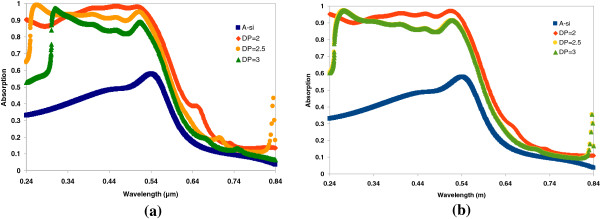
**The optical absorption enhancement on thickness of 100-nm a-Si:H thin film.** The film is with an array of (**a**) 100 × 100 × 100 nm cubic blocks; (**b**) both height and diameter of 100-nm cylinders.

The effects of the ratios of the areas of the nano-particle to the unit cell to the optical absorption enhancement are investigated with the FDTD simulations. In these simulations, the periodicities of the unit cell are varied, and meanwhile, the thickness of the a-Si:H thin film is 100 nm. The features of the nano-block and nano-cylinder are kept as constants, too. For example, the size of nano-block is 100 × 100 × 100 nm (*D* = 100 nm), the radius and height for the nano-cylinder are 50 nm (*D* = 2 × 50 = 100 nm) and 100 nm, respectively. The optical absorption spectra of periodicities of the unit cell of 200 nm (DP = 2), 250 nm (DP = 2.5), and 300 nm (DP = 3) are shown in Figure 3. These plots show that the periodicity of 200 nm has better absorption enhancement than periodicities of 250 and 300 nm for both types (block and cylinder) of particles. The LT effect is seen in a case that very little of the surface of the thin film was covered by the array of nano-cylinders; for periodicity of 300 nm, the ratio of the particle size to the unit area is only 11% (DP = 300 nm).

**Figure 4:**
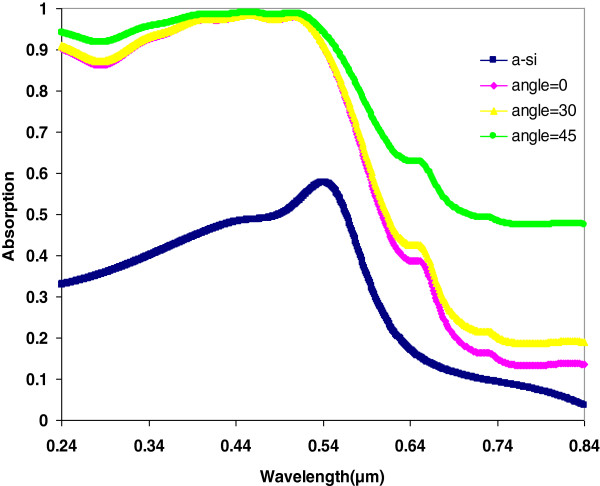
**Optical absorption of 100-nm thick a-Si:H thin film.** The film is with metallic nano-blocks for the incident light at various incident angles.

The role of the incident angle of the light in the LT is investigated, too. We keep the azimuthally angle *φ* to zero and vary the incident angle. The optical absorption enhancement of the incident angles of 0°, 30°, and 45° are shown in Figure 4. The FDTD simulations show that the absorption efficiency of the incident angle of 45° is highest over the spectra, and the enhancement in the red light region is significant. This can be understood as the surface plasmon can be induced higher efficiently by the incident light with a bigger angle (see Equation 1).

## Results and discussion

Optical absorption in thin a-Si:H film enhanced by metallic nano-particles was investigated by simulations. The investigation of the scattering of metallic spherical particles shows that it is possible to provide larger scattering cross-section than geometry and absorption cross-sections for particles with a diameter of 100 nm or bigger. The scattering of metallic nano-particles makes the light travel in the thin film in a longer path; therefore, higher optical absorption occurs due to more opportunities of the light to interact with the medium. Besides the scattering, the metallic nano-particles convert part of the incident light to surface plasmons, which propagate on the surface of the thin film and in the thin film. The FDTD simulations of the metallic nano-particles show that the absorption of the red spectrum is enhanced by the nano-particles (nano-blocks and nano-cylinders). For the height of 100 nm, particles have significant enhancement for red-light absorption.

## Conclusions

Our study shows that the dominant enhancement effect comes from the surface plasmon resonance while the scattering contributed partial enhancement, and it is the main reason of using metallic particles which not only induce surface plasmons but also scatter incident light. We also study the optical absorption enhancement for incident light with an angle. It shows that the 45° incident light has better enhancement in the red light; this could be mainly because the coupling efficiency of light to the surface plasmons is higher due to the wave vector of the surface plasmons as described in Equation 1. Our study indicates that the optical absorption can be enhanced in the red spectrum with metallic particles of a high coupling efficiency from light to surface plasmon. In order to achieve this, one has to carefully select the type of metal and the structure and size of the particles.

## Abbreviations

a-Si:H: Hydrogenated amorphous silicon; LT: Light trapping; FDTD: Finite difference time domain; PML: Perfect matched layer.

## Competing interests

The authors declare that they have no competing interests.

## Authors’ contributions

YT did most of the simulations, plots, and the manuscripts. BV input many ideas on the structures in the simulations and did some plots. All authors read and approved the final manuscript.

## Authors’ information

YT works on the nano-device development at NCCU since 2006; BV is the director of CREST and works on nanotechnologies and many other areas.
